# Selections and Abstracts

**Published:** 1915-01

**Authors:** 


### SELECTIONS AND ABSTRACTS

### EXPERIENCES OF THE NEW YORK HEALTH DEPARTMENT IN TYPHOID IMMUNIZATION.

By L. I. Harris, M. D., Assistant to Director, Bureau of Infectious diseases.

Case Reports by Morris L. Ogan, M. D., Chief, Division of Typhoid Fever, New York.

Preventive medicine, despite its brilliant advances in recent days, has met momentary checks, when, as happens from time to time, some apparent instances of its failure to protect from disease have come to light. That element of our lay population which suffers from constitutional timidity and which can be induced only with difficulty, if at all, to adopt the teachings of this newest and most helpful branch of medicine, jumps eagerly at these apparent examples of failure. It makes them serve as justification for relapsing into the helpless and fatalistic attitude toward disease which has come down through the centuries from the dark ages. These particular instances of the so-called miscarriages of prophylactic medicine are trumpeted far and wide and marshaled to serve as evidence against its value, long years after impartial scientists have disproved all criticism and vindicated the signal virtues of this newest department of medical science.

Error and hasty and mistaken conclusions die as hard today as ever in the history of scientific progress. In common with every method of treatment that has ever been inaugurated, antityphoid prophylactic treatment has been opposed at each step of its progress by criticism which has sometimes been ignorant, or unscholarly, or malicious, or even all of these. Russell has already paid his compliments to these groups. This study of antityphoid immunization is therefore offered to answer unsound criticism as recently expressed in connection with a series of cases that received conspicuous notices in the New York press. Also it aims to give to those general practitioners who have read the necessarily insufficient and confused comments on these cases in the lay press and even in some of the medical journals a knowledge of facts interpreted scientifically, that they in their praiseworthy office as teachers be reassured and reinforced in their belief in the value of this protective measure, and may impart with renewed enthusiasm and conviction the full measure of their belief to their pupils —the public.

THE EARLY HISTORY OF ANTITYPHOID VACCINATION.

Almost simultaneously in 1896, Pfeiffer and Kolle in Germany, and Wright, of Netley, England, published the results of eyperiments in the prevention of typhoid fever by vaccination with Bacillus typhosus killed by heat.

For several years preceding, a group of workers including Dunbar, Frankel, Simmonds and Stern, had been occupied with the problems of immunology in typhoid fever. Wright made an extensive application of these experiments, vaccinating large groups of men in the British army. His results were not as favorable as had been expected, and the procedure met a setback until Leishman, who had been working with Wright, again essayed the use of this prophylactic measure on a large scale on the men in the Indian service of the British army. He had concluded that their failure to achieve the success they had anticipated in their first trials was due to a superheated vaccine. This fault was corrected and Leishman's work gave a new impetus to those interested in the subject.

When, subsequently, Wright announced his theory of the negative phase, and the increased risk of infection with typhoid fever, during the period of this phase, progress for a time was again checked. The cumulative, practical experience of Leishman, however, enabled him to refute this theory most emphatically, and to prove conclusively that whatever force it had was more theoretic than real, and "that the most important evidence must come from actual practice, rather than from laboratory experiment." Indeed, Pfeiffer and Friedberger disproved this in laboratory tests on guinea-pigs, as did Leishman in 100 cases at the Baring asylum. Wright himself since 1909 has receded from this position to a very great degree.

In 1911, Major F. F. Russell, than whom there is no more competent authority on this subject in this country, had so far advanced its use among 20,000 men in the United States army that he could assert of those using it that "we knew that the preparation we were using was harmless; we knew too that it caused few severe reactions in healthy persons, and that no vaccination, however severe the immediate reaction may have been, had been followed by permanent injury to the individual; and further, that by all laboratory tests the immunity conferred was identical with and equal to that remaining after typhoid fever." The brilliancy of the results achieved by Major Russell at San Antonio, and subsequently, on an even larger scale in the army, results which now have become almost classical, led to the enactment of the compulsory antityphoid vaccination law by the United States government.

In France, in 1913, a similar law was passed, making vaccination against typhoid fever compulsory in the French army.

Fully 85 per cent of the men in the Indian service of the

British army have been vaccinated, although no legal compulsion has been employed by the British government.

In the years that have elapsed since 1896, the evidence of the value of this measure is undoubted, and has grown overwhelmingly; so that when the death-rate in the United States was 16.5 per hundred thousand, that in the United States army during 1912 was 0 per hundred thousand. Further examples selected from this accumulated testimony, as they bear on the analysis of our cases, will follow. Well has Russell said, "Our experience since 1909 has left no doubt about the success of antityphoid vaccination in the army and navy. The measure has come to stay."

THE VACCINE—DESCRIPTION AND MODE OF PREPARATION.

The prophylactic injections that have been in use have been differently prepared by the various men who have administered them: Leishman in the British army and Russell in America, as well as most other Americans who have employed this procedure, have used a single strain (the Rawlings strain) which is of low virulence. The French prepared a polyvalent vaccine of as many as ten strains, consisting mainly of those found in the neighborhood where an outbreak of typhoid exists. Even in America, some, like Hunt, in Troy, Pa., have employed a polyvalent vaccine in which has been included a B. paratyphosus A, and B. Paratyphosus B of Gaertner. Needless confusion in the minds of those not familiar with this subject has been caused by the description of these and a variety of other preparations of vaccine.

Metchnikoff and his pupil, Besredka, for instance, have used a vaccine consisting of an emulsion of living typhoid bacilli sensitized by antityphoid serum. Up to 1913 this vaccine, first employed on chimpanzees, was injected into 800 persons in an institution, without any case developing after its use. One soldier in Africa so treated died of typhoid fever, but Metchnikoff explains this as being due to a dose that was entirely too small. Also, forty-eight patients with pronounced typhoid fever were treated with this vaccine. But the concensus of opinion is that aside from the danger of producing carriers, it is far safer to use the sterilized single Rawlings strain of low virulence, which has proved so brilliantly effective in England and the United States.

Chantemesse has produced an antityphoid serum obtained from horses, which has not been approved of by others.

The New York Board of Health vaccine conforms in every particular but one to the army vaccine; the exception being that phenol (carbolic acid) has been substituted for trikresol as a preservative, because of the lessened pain caused by its injection.

The culture used by the Department of Health is prepared at its Research Laboratory and is made as follows:

"A laboratory culture of typhoid bacilli which has lost much of its virulence through long artificial cultivation is used. Large surfaces of agar in Blake bottles are inoculated from fresh agar cultures. After twenty-four hours' growth at 37 C. the bacteria are washed from the surface of the agar with normal salt solution. The suspension is then standardized by counting the bacilli by the Wright method. This is done by mixing an equal part of blood and the bacterial suspension. Smears are made from this mixture and stained. The number of bacilli and red cells are then counted under the microscope in about twenty-five fields. When the proportion between the two has been determined, the number of red cells per c. c. being known, the number of bacilli per c. c. can then be estimated; this suspension is then heated one hour at 56 C., to kill the bacilli. After heating, the sterility of the suspension is tested by inoculating generous amounts into media and incubating these under aerobic and anaerobic conditions. If no growth occurs, 0.25 per cent of carbolic acid is added to the suspension which is diluted with 0.25 per cent carbolic acid in normal saline solution so that one c. c. contains the appropriate dose. It is then bottled for distribution."

### CASE REPORTS AND COMMENTS.

Whenever we add to the sum of our knowledge and experience, we describe a larger circle to embrace the new as well as the old, and so in this study, even if we contribute little that is essentially new, we believe it well in what follows to include a review of much that has gone before, so that a comprehensive and better correlated picture may the more vividly appear.

In the following are presented the histories of the cases whose atypical features offer several problems of interest, and which have prompted this survey of the work of the New York Department of Health in the field of antityphoid prophylactic treatment.

It will be noted that they fall into four classes:

Class 1. In which death has been attributed to antityphoid inoculation, and was really due to another cause, the prophylactic treatment being a mere coincidence.

Class 2. Severe reactions within a few hours after treatment.

Class 3. In which immunization was partial and insufficient, the patients having been long exposed and already in the stage of incubation when treatment was begun.

Class 4. In which a complete course of prophylactic treatment failed to give immunity, and was followed after a relatively short interval by an attack of the disease.

CLASS 1. COINCIDENCES.

CASE 1.—C. P., aged 25, a member of the National Guard, received three injections of antityphoid vaccine in January, 1914. He reacted very moderately to these injections, and for several weeks thereafter continued in good health. Early in March, that is, two months after immunization, he became ill, and the physician who attended him diagnosed his illness as influenza. Just before his death, which occurred March 30, 1914, another physician who was called in diagnosed his case as typhoid fever, and so certified in the death certificate he issued.

Acting on the protest of the surgeon of the regiment to which the patient had been attached, the Department of Health refused to accept the death certificate, and a necropsy was therefore ordered.

April 2 the coroner's physician performed the necropsy, and established the absence of typhoid lesions. The heart was found to be the seat of a malignant ulcerative endocarditis. The skin showed petechiæ, and septic infarcts were found in various parts of the body, the kidneys particularly.

This case, in which malignant endocarditis ran such a deceptive clinical course that the physicians in attendance were led to believe they were dealing first with influenza and then with typhoid fever is of a type not unfamiliar in clinical medicine and which frequently remains obscure as to diagnosis until it reaches the necropsy table. Such an erroneous charge against typhoid vaccination as this needs no further refutation. The entire lack of logical sequence between vaccination and the ultimate cause of death is clearly evident.

It is reminiscent, however, of the early history of small-pox vaccination, in which, in the case reported by Broeckerhoff, the Jennerian operation was followed by an illness which was held to be small-pox, and because it resulted in death, seemed, until cleared up by necropsy, an aspersion on vaccination. Death in this instance proved to be due to hemorrhage from a typhoid ulcer, and not to the use of vaccine. So once again, it is interesting to note how consistently history repeats itself.

### CLASS 2. SEVERE REACTIONS.

CASE 2.—A clerk in one of the divisions of the Department of Health was injected one afternoon with antityphoid vaccine, and it was noticed that on withdrawal of the needle a free stream of dark blood followed. This was interpreted to be due to the puncture of a vein. Not more than half an hour later, he was seized with sudden faintness, his skin became pale and clammy, he complained of headache, showed dulness of intellect, and temperature of 103. These symptoms did not last more than a few hours, and the next day he was up and about ready to return to work. The entrance of the vaccine directly into the venous stream was held accountable for the train of symptoms; but not lasting or serious results remained.

Occasionally an urticarial rash on the chest and abdomen, lasting for a week, has been reported, as has also herpes facialis following these injections.

HISTORIES OF CASES 3, 4, 5, 6 AND 7.

INTRODUCTION.—Mr. M. arrived at his home in Brooklyn from a business trip through the Southern states, April 23, with a history of illness of ten days' duration. Mr. M.'s physician submitted a specimen of his blood for a Widal test to the New York Department of Health, and on April 25 he was informed that it gave a positive Widal reaction.

At noontime, May 3, in compliance with Mr. M.'s request, the physician administered an immunizing dose of Department of Health vaccine to each of the four presumably well members of the family, consisting of his wife and three children, Ruth, Herbert and Raymond. May 16, Mr. M. died. Mrs. M.'s sister, a Mrs. R., who was present when the prophylactic injections were administered, also received one.

CASE 3.—Mrs. R., the sister-in-law of Mr. M., returned to her home in New York, a distance of at least five miles, and subsequently stated that she had been feeling "very sick" that night. She called no physician to see her. No temperature record or other competent observations were made at the time. She was entirely well in two or three days and able to perform her regular duties. Her case is mentioned because, in association with the four that follow, it served to inspire the criticism and detraction of a few organs of the lay press and, forsooth, of a medical journal, too. In common with Herbert M. and Ruth M., whom Mrs. R. brought to her New York home, five miles distant, directly after they had received prophylactic injections, she made a journey, the length and fatiguing nature of which were contra-indicated after immunizing treatment.

CASES 4, 5, 6 AND 7.—These four cases are those of Mrs. and again in the next, because they illustrate both the phenomenon of a severe reaction, and the futility of preventive inoculation when administered during the period of incubation.

CASE: 4, 5, 6 AND 7.—These four cases are those of Mrs. M. and her three children, two of whom, Ruth, aged 4½, and Raymond, aged 6 years, made the long trip to New York with their aunt. Within twelve hours after their injection all are reported as having been "very sick," but temperatures were not taken, and no more precise and detailed clinical notes are on record as to this particular period than is contained in the foregoing incomplete statement of symptoms. Mrs. M. felt well again within two or three days, and continued nursing her husband. The other children also showed but a temporarily severe reaction, with an interval of comparative moderation in symptoms, preceding the conditions to be outlined in that part of the history which is assigned for description in the next class.

In these five cases in which severe reactions were reported to the family physician by the patients themselves, several factors were operative. First, the comparatively long journey to New York made by three of those treated; secondly as will appear from the subsequent history, the M. family so treated were not in good health and probably then already in the period of incubation of the disease.

Of this class, characterized by severe reactions that ensue at any time, either immediately after the injection of an immunizing dose of vaccine, or from twelve to forty-eight hours later, much may be said by way of impartial critical comment.

Practically our entire navy has been vaccinated without a single serious result or casualty being noted; and less than 1 per cent of the men were obliged to rest in bed or be excused from duty as a result of the reaction that followed.

Russell in 128,903 cases observed severe reactions as follows:

After the first dose, 0.3 per cent in 45,680.

After the second dose, 0.2 per cent in 44,321.

After the third dose, 0.1 per cent in 38,902.

Authoritative writers define a severe reaction as one characterized by a temperature of from 103 to 104, malaise, vomiting, diarrhea and chills.

Spooner, in 400 cases, noted severe reactions in 4 per cent; moderate reactions, consisting of malaise, headache and a temperature of not above 100.5 in 10 per cent, and very slight or absent reactions in 86 per cent.

Hachtel and Stoner of Baltimore immunized 1,326 persons with an absence of symptoms of distinct reaction in 1,160 or over 87 per cent.

Malaise appeared in 775.

Headache appeared in 835.

Muscular pains appeared in 106.

Nausea and vomiting appeared in 60.

Chills appeared in 57.

Temperature rise appeared in 884.

A study of the temperatures gave the following results:

Temperature normal to 101 in 785.

Temperature from 101 to 103 in 82.

Temperature over 103 in 17.

In 1,945 persons on whom they employed a monovalent strain of vaccine, they found that 95.8 per cent had normal temperature or only a slight rise; in ninety-nine on whom the polyvalent vaccine was used, a normal or slight elevation of temperature was observed in only 80.7 per cent. In other words, severe reactions seem more likely to follow a polyvalent vaccine.

Ravenel, of Wisconsin, vaccinated 1,900 and noted mild reactions in all but two, who after receiving their third injections had drunk heavily.

Weston, in an experience with 898 persons to whom he administered prophylactic treatment, found a severe reaction after the first dose in 883; a severe reaction after the second dose in 880, and a severe reaction after the third dose in 895.

Of those who received this treatment in the French army, at home and in Africa, severe reactions were noted only in those who were ill or fatigued at the time of the injection, this, despite the fact that a polyvalent vaccine (consisting of ten strains) was employed.

Dr. John W. Brannen, using the New York Department of Health vaccine at Bellevue Hospital on 200 nurses, fifty of the physicians on the house staff, and also on a few male patients in his private practice, reports that of these, only three nurses asked to be relieved of their duties for one day, several interns had a rise of temperature as high as 102, and one doctor had a temperature of 103, with headache and abdominal pain after each of two doses.

Rukke, of St. Louis, vaccinating 11,007 cases, giving 33,-021 injections in all, noted severe reactions in eighty-seven who, in conformity with practice in the United States army, had received simultaneous small-pox vaccinations. Of these eighty-seven, 50 per cent had vaccinia reactions or reaction from measles or other infectious diseases. Only one case of musculo-spiral neuritis developed in an officer as the result of trauma to a nerve. He asserts, on the basis of experience in the families of officers living in the camps, that children bear the inoculation well, even at as early an age as 3 years.

Pensuti, using higher doses of vaccine than those generally employed, vaccinated eight children (from 7 to 12 years of age), without subsequent trouble.

All these citations are to show that the occasional occurrence of a severe reaction has been acknowledged and understood by all competent authorities. The severity of these reactions is determined, as already mentioned, by debility from illness, (tuberculosis, etc.) marked fatigue, alcoholism or undue exertion in those subjected to prophylactic treatment. In other instances, injection of the vaccine directly into a vein or its transmuscular administration, has accounted for very marked reactions.

In civil, as distinguished from military communities, whenever a case in which a severe reaction has appeared has been given publicity, it is usually a professional brother who helps spread misinformation with the benevolent suggestion that a contaminated or improperly prepared vaccine has been responsible for such serious result. This charge, if true, is indeed a grave one. Let us examine it: The heating of the suspension of bacilli that is used in preparing the vaccine, at 56 C. (132.8 F.) and the subsequent inoculations into mediums incubated under aerobic and anaerobic conditions to test the sterility of the suspension, together with the final addition of 0.25 per cent phenol, would surely preclude contamination. The only organisms that might then possibly be present as contaminating ones, would be the tetanus bacillus or staphylococcus. The presence of these organisms could surely be recognized—the tetanus organism by the characteristic symptomatology resulting from its action, and the staphylococcus by a localized infection at the site of injection. From the history of the cases thus far given, and as it subsequently appears, it will be clearly apparent that neither of these contaminations of the vaccine were present.

All those who are acquainted with the character and the organization of the Research Laboratory of the New York Department of Health, will feel assured that the vaccine is prepared with every caution and safeguard against contamination. Moreover, Drs. Curran, W. S. Hubbard, F. Harnden, C. R. Love and Dudley, of Brooklyn, employed vaccine of the same stock as the foregoing on members of their own families and on private patients without a single severe reaction or untoward result.

CLASS 3. PARTIAL AND INSUFFICIENT IMMUNIZATION.

The history of Cases 4, 5, 6 and 7 is resumed under this classification because after the severe reaction caused by the single injection, which each of these patients received, and the short interval of freedom from complaint that ensued, the symptoms of typhoid fever made their appearance in each case.

CASE 5.—Ruth, aged 4½ years, became ill May 7, and was brought back to her home in Brooklyn from New York, by her aunt. The temperature became high (about 104) and hovered about that point for a number of days, while signs of severe meningeal irritation supervened, that is, marked opisthotonos, stupor, muscular rigidity and loss of sphincter control—justifying the name frequently applied to typhoid, Nervenfieber.

Lumbar puncture, performed May 20, proved negative for meningococci, and the spinal fluid was clear and under considerable pressure. A blood count at this time gave the following results: Polymorphonuclears, 41 per cent; small lymphocytes, 47 per cent; large lymphocytes, 9 per cent, mononuclears, 3 per cent.

The Widal test showed agglutination in dilution of 1:60 in ten minutes; 1:120 in ten minutes; partial agglutination, 1:500 in ten minutes; complete in thirty minutes; partial agglutination, 1:1,000 in thirty minutes; complete in one hour.

May 22, an examination of the feces showed typhoid bacilli. There was no doubt therefore that this was a case of typhoid fever which became manifest shortly after a single injection of antityphoid vaccine. The patient subsequently made a complete recovery.

Cases 6 and 7.—The other children, Raymond, aged 6 years, and Herbert, aged 11 years, also developed symptoms of typhoid fever several days after they had recovered from their severe reactions. Raymond's case ran a brief and uneventful course. Herbert had a moderately severe attack of several weeks' duration with a relapse. These two also made complete recoveries.

Case 4.—Mrs. M., the mother, who had been assiduously nursing her husband until the time of his death, May 16, and then had similarly cared for her children, kept at her work even when initial symptoms of typhoid fever had made their appearance. Like so many ambulatory cases, the date of onset of her illness could not be definitely established. Despite malaise and an increasing feeling of illness she continued her household duties and the care of her children until June 13, when her physician reported her ill with typhoid fever. On June 15 she died of intestinal hemorrhage.

Clearly this unfortunate family continuously and intimately in contact with a typhoid fever case for a number of days, before a single vaccine injection was attempted, had incubated the typhoid bacilli too long to make effective the antagonistic and immunizing power of antityphoid vaccination.

(To be Continued)

### EXAMINATION OF CANDIDATES FOR ASSISTANT SURGEON.

TREASURY DEPARTMENT.

United States Public Health Service.

Washington, January 11, 1915.

Boards of commissioned medical officers will be convened to meet at the Bureau of Public Health Service, 3 "B" Street, S. E., Washington, D. C., and at the Marine Hospitals of Boston, Mass., New York, N. Y. Chicago, Ill., St. Louis, Mo.; Louisville, Ky., New Orleans, La., and San Francisco, Cal., on Monday, March 8, 1915, at 10 o'clock a. m., for the purpose of examining candidates for admission to the grade of assistant surgeon in the Public Health Service, when applications for examination at these stations are received in the Bureau.

Candidates must be between 23 and 32 years of age, graduates of a reputable medical college, and must furnish testimonials from two responsible persons as to their professional and moral character. Service in hospitals for the insane or experience in the detection of mental diseases will be considered and credit given in the ,examination. Candidates must have had one year's hospital experience or two years' professional work.

Candidates must be not less than 5 feet, 4 inches, nor more than 6 feet, 2 inches in height.

The following is the usual order of the examinations: 1, physical; 2, oral; 3, written; 4, clinical.

In addition to the physical examination, candidates are required to certify that they believe themselves free from any ailment which would disqualify them for service in any climate and that they will serve wherever assigned to duty.

The examinations are chiefly in writing, and begin with a short autobiography of the candidate. The remainder of the written exercise consists of examination in the various branches of medicine, surgery and hygiene.

The oral examination includes subjects of preliminary education, history, literature and natural sciences.

The clinical examination is conducted at a hospital.

The examination usually covers a period of about ten days.

Successful candidates will be numbered according to their attainments on examination, and will be commissioned in the same order. They will receive early appointments.

After four years' service, assistant surgeons are entitled to examination for promotion to the grade of passed assistant surgeon.

Assistant surgeons receive $2,000, passed assistant surgeons $2,400, surgeons, $3,000, senior surgeons, $3,500, and assistant surgeons general $4,000 a year. When quarters are not provided, commutation at the rate of $30, $40 and $50 a month, according to the grade, is allowed.

All grades receive longevity pay, 10 per cent in addition to the regular salary for every five years up to 40 per cent after 20 years' service.

The tenure of office is permanent. Officers traveling under orders are allowed actual expenses.

For invitation to appear before the board of examiners, address "Surgeon-General, Public Health Service, Washington, D. C."

---

### FOREWORD.

### From The Medical Pickwick.

A new Journal, devoted to the humorous and human side of our profession, launched at a time when more than half a hemisphere is displaying an "inhuman lack of humor" in an unthinkable war, is certainly a novelty and may seem to many an anomaly. Yet, if we consider the fatalism of the present war, the saignee a blanc dreaded, if not predicted, by the wise Bismarck, a horror which dissipates our fondest dreai and projects into space, should not a periodical of this kind, designed to be, each page of it, a brief for sanity and humanity, receive the cordial support of our profession? For in nine-tenths of medical practice, a fund of sanity and humor, the sign and symbol of large-minded effectiveness, is of vastly greater moment than the pedantic machine-made "efficiency" which is the word of ambition at the present hour.

The chief end of the soldier is to destroy the enemy. The chief end of the physician is to save life, no matter whose. Yet medicine is undoubtedly one of the most disagreeable of the professions, full of petty cliques and clans, active dislikes and hatreds, professional jealousies and ugly controversies, some of which have extended over vast periods of time, and which only the modern spirit, which teaches that toleration should not be extended to the intolerable, has tended to lessen. The soldier, with the priest, is the solitary survivor of the feudal system in modern life. The whole law of his being is summed up in two words: Obey orders. If it should be even decreed that he march into certain death by the book, march he must, even though "someone had blundered." "The main thing for a soldier," says Stevenson, "is to be silent, and the chief of his virtues never to complain." The physician, on the other hand, has, in his capacity of life-saver, to deal with the most disagreeable facts in existence, the pains of birth and death and all intermediate phases of life in which the human frame is tortured, and even twisted out of shape, by injury or disease. In certain periods it was part of his professional dignity to assume, if not to effect, a preposterous omniscience, pompous infallibility, which he invariably challenged in his fellows when not challenged by them. From the moment that physicians begin to accept fees for their services, the profession had already been "commercialized," in the sense of creating an atmosphere of competition along undesirable lines. The recent phases are only a detail of the wholesale capitalization and commercialization of all modern industry. We war upon proprietary preparations and patented nostrums, but the great eighteenth century physicians had, each one of them, certain pet prescriptions, which they kept strictly private, letting it be known that they were in possession of all but infallible methods of handling disease. This set the vogue for the great army of quacks who flourished in the age of periwigs. It is one of the tritest of modern observations that quackery thrives best where science is in the highest ascendancy. The top-heavy spirit of infallibility which is sometimes affected by the votaries of science gives the charlatan his opening with poor human nature, ever distrustful of the "superior persons"—this and the fact that, for the great majority of physicians who are honest and sincere with themselves, much of therapeutics is still experimental and empirical. Here, as Abraham Flexner observes, "the very candor of scientific medicine gives the charlatan his chance, for, just where the scientific physician admits his inadequacy, the charlatan is most positive." A well known modern aphorism asserts that every fashionable physician has in him something of the quack. All these things have made for the disagreeable in medical practice, and one object of our local medical societies, national associations, international congresses, banquets, Festschriften, loving cups, medical medals and purses is to smooth down these sharp corners. Billroth, looking over an old collection of medical medals, shrewdly remarked that not a single one of them had been struck off to commemorate anything more than respectable mediocrity. It is only of late years that memorial medals and tablets have been fashioned in honor of the truly great. Never has scientific merit been more duly recognized than now. Yet we find that the great practitioners of the Sydenham type, the true family doctors, have had little in common with the great clinical teachers, the Addisons, the Frerichs and the Charcots, but were unaffected, humorous people, studying the patient as well as his disease, and knowing as much of human nature as of the insides of books.

"What an ornament and safeguard is humor!" said Emerson of Walter Scott. "Far better than wit for a poet and writer. It is genius itself, and so defends from the insanities."

To cultivate this genial, humorous spirit in our profession, to consider medicine in a Pickwickian sense, is the object of this journal. The editor's aim, we are assured, will be to make it exclusively an organ of the literary and cultural side of our profession. Pure science, intensive research in medical history, dry discussion of technical matters, will find no place here; but there will be always room for the merry quip, the effective anecdote, the happy allusion, the apposite citation, the "Pickwick Medical Society," and other humoresques; a page or so each month in memory of the great medical men who have cultivated the humaniora; a series of excerpts from secular literature illustrating the cultural aspects of medicine through the ages; a place for poems, fiction, satire, travesty; and for the physician's uplift, the sursum corda, a corner for those short poems which Goethe defined as the only verse which is truly inspired, the Gelegenheitsgedicht. The editor can be relied upon to steer a course equidistant from silly squeamishness, and all that comes under the ban of Roscommon's sarcasm.

"For want of decency is want of sense."

The genius of an entire race is in that line, and no infraction of this plan will be tolerated in these pages.

At a time when every morning or evening newspaper is a bulletin of

"Night's darkness, and blood-dripping wounds,
And psalms of the dead."

it is difficult for many of us to see things in a humorous light. We of the Anglo-Saxon race, who, next to the honor of being born in this country, regard our Anglo-Saxon kinship as the most priceless spiritual treasure which we possess, cannot but view the severance between England and German with sadness. Goethe said, in his conversations with Eckermann: "In my poetry I have never shammed (nie affektirt). I have never uttered anything which I have not experienced, and which has not urged me to production. I have only composed lovesongs when I have loved. How could I write songs of hatred without hating! And between ourselves, I did not hate the French, although I thanked God that we were freed from them. How could I, to whom civilization and barbarism are the only distinctions of importance, hate a nation which is among the most civilized on earth and to which I owe a great part of my own cultivation? Altogether, national hatred is something peculiar. You will find it the strongest and most violent at the lowest plane of culture. But there is a plane where it vanishes altogether, and where one stands to a certain extent above nations, and feels the weal and woe of a neighboring people, as if it had been one's own. This degree of culture was conformable to my own nature, and I had become strengthened in it long before I had reached my sixtieth year." Beside these majestic utterances of a noble mind let us place this sentence of Renan, luminous with intelligence of the highest order: "Au desus de la race, il y a l'humanite: ou, si l'on veut, lo raison."

In time of war, this journal is dedicated to the cause of peace and comity, good humor and good fellowship among medical men, in the words of one who is not only Nestor of our profession, but to us, the most distinguished German-American citizen living, Dr. Jacobi: "Aims, methods and persistency are common to the medical profession of all countries. On its flag is inscribed what should be the life rule of all nations: Fraternity and solidarity.--F. H. G.

